# Relevance of indirect comparisons in the German early benefit assessment and in comparison to HTA processes in England, France and Scotland

**DOI:** 10.1186/s13561-014-0031-5

**Published:** 2014-11-30

**Authors:** Andrea Lebioda, David Gasche, Franz-Werner Dippel, Karlheinz Theobald, Stefan Plantör

**Affiliations:** Health Economics & Outcomes Research, Real World Evidence Solutions, IMS Health GmbH & Co. OHG, Munich, Germany; Health Economics & Outcomes Research, Real World Evidence Solutions, IMS Health, S.A., Barcelona, Spain; Sanofi-Aventis Deutschland GmbH, Evidence based Medicine & Health Economy, Berlin, Germany; Sanofi-Aventis Deutschland GmbH, Evidence based Medicine & Health Economy, Frankfurt am Main, Germany

**Keywords:** AMNOG, Early benefit assessment, HAS, Indirect comparison, IQWiG, NICE, I18

## Abstract

Early benefit assessment in Germany under the legislative framework of AMNOG (*Arzneimittelmarktneuordnungsgesetz*) requires direct comparisons of the new drug with appropriate comparators determined by the Federal Joint Committee (G-BA). In case no head-to-head studies are available for direct comparisons, the submission of indirect comparisons is permitted to assess the additional benefit of the new drug. However, the Institute for Quality and Efficiency in Health Care (IQWiG) states a clear preference for head-to-head trials and defines strict requirements for indirect comparisons to be considered in the benefit assessment. Similar requirements also exist in other countries with mandatory health technology assessments (HTA), like France, England and Scotland. Our evaluation shows that a comparison of the different HTA regarding indirect comparisons is difficult. Overall, external preconditions and methodological requirements are demanding and hardly to fulfill by pharmaceutical companies for implementation of indirect comparisons in early benefit assessment. The determination of the appropriate comparators, outcomes, patient subgroups and study choice are the main target within indirect comparisons for the future. To compare and assess submitted indirect comparisons it would be desirable that a transparent process was established, including the mandatory publication of HTA-reports within Europe and international guidelines, accepted by a large number of HTA-agencies.

## Background

Since the AMNOG came into force in 2011, all pharmaceutical companies have to present clinical evidence to proof an additional benefit of new substances compared to appropriate comparators. The early benefit assessment in Germany under AMNOG requires direct comparisons with one of the appropriate comparators determined by the G-BA for each therapeutic indication to provide clinical evidence of their product. Since 2013 the G-BA allows more than one appropriate comparator and it is up to the pharmaceutical company to choose one of these. In case no head-to-head studies are available, the submission of indirect comparisons is permitted to assess the additional benefit of the new drug. However, the IQWiG states a clear preference for head-to-head trials and defines strict requirements for indirect comparisons to be considered in the benefit assessment. Similar requirements are also existent in other countries with mandatory HTA, like England, France and Scotland. Besides, a joint development of methodological approaches between different European countries began. This is necessary as statisticians and scientists face the same HTA-problems.

### Definition and methodology of indirect comparisons

The gold-standard to compare efficacy, effectiveness and safety of interventions in healthcare are randomized, active controlled trials. The need of an indirect comparison arises when no head-to-head trials are available to perform a direct comparison of two different interventions. Indirect comparisons can be performed as adjusted indirect comparisons, unadjusted indirect comparisons and mixed-treatment-comparisons (MTC). Despite of the specific methodology, a common comparator is required in adjusted indirect comparisons [1-4].

#### Adjusted indirect comparison

**Figure 1:**
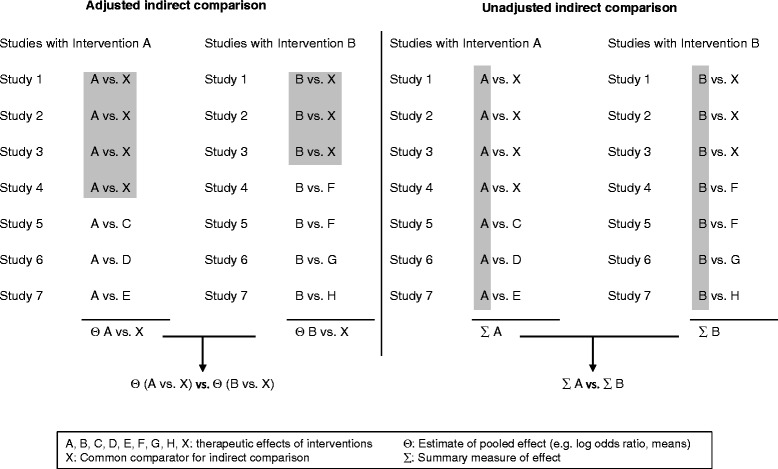
**Adjusted and unadjusted indirect comparisons. **Source: [3,5], adapted.

Bucher’s method is the most common and accepted approach for an adjusted indirect comparison of two interventions A vs. B [4]. Adjusted indirect comparisons preserve the strength of randomization by comparing the (pooled) relative effect estimate of intervention A vs. X with the (pooled) relative effect estimate of intervention B vs. X (X as common comparator), as in Figure 1 with common comparator X. Placebo is often used as a common comparator [2-5].

#### Unadjusted indirect comparison

An unadjusted indirect comparison is defined as the naïve use of single study arms to combine study data as if coming from a single large trial. A summary effect is computed for all study arms involving intervention A and compared to a summary effect for all study arms including intervention B. These two summary measures (cohorts) of the effect of intervention A and intervention B will be compared to establish the relative effectiveness of intervention A to intervention B. Data of comparative arms will not be used, as shown in Figure 1. Thereby the randomized nature of individual trials is compromised. It is recommended to avoid unadjusted indirect comparisons, as they are seen as an inadequate method [2,3,5-7].

#### Mixed-treatment-comparisons (MTC)

**Figure 2:**
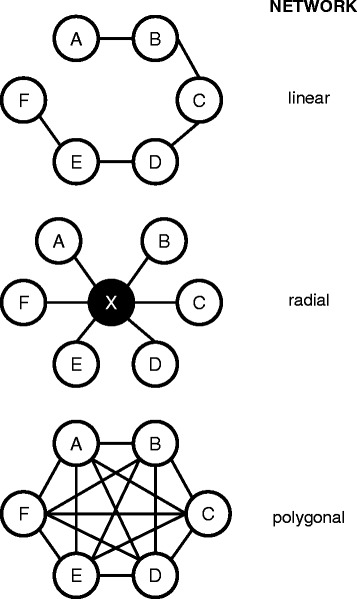
**Networks in MTC and their geometrical structure. **Source: [5], adapted. Letter A to F are representing trials, X is common comparator.

In the last years, an increased performance of MTC could be observed in the publications of indirect comparisons. MTC are also known as network meta-analyses. They compare more than two different treatments simultaneously. MTC allow the combination of indirect and direct comparisons, incorporation of multiple common comparators and the use of a larger overall number of trials [3,5,8,9]. Within a MTC, there are often used networks with geometrical structures. Possible structures of networks are shown in Figure 2.

#### Bayesian and frequentist approach

Bayesian approaches are common methods within MTC as direct and indirect evidence can be combined. The Bayesian approach uses point estimates and credibility intervals to give easy and uncontroversial results in probability theory as traditional statistical methods and is therefore more flexible. In general, Bayesian methods use the Bayes-Theorem to interpret the unknown estimate by converting outcomes of one or more trials of a prior probability distribution in a posterior distribution – likelihood estimation. External evidence (also from non-RCTs) and the form in which conclusions are drawn are the contributors for decision making [5,6,10-12].

Commonly, the frequentist approach is used in direct comparisons [6,12], but as researches show, the use in indirect comparisons, i.e. MTC, is rising. The parameter of interest is a fixed state of nature as in contrary to a Bayesian approach [10]. The frequentist approach is divided into fixed and random effect methods. A fixed effect model aggregates outcomes of different trials with the assumption, that all estimated effects are similar and differences are coincidences. Uncertainties of the outcomes can be affected only by the individual trial [5,6,12]. A random effects model calculates with pooled outcomes with regard to effect differences in different trials. The variation of effects within trials and between trials is included in the model [5,12].

#### Individual participant/patient data (IPD)

The use of IPD is possible within direct and indirect comparisons as well as within MTC, which is very common, in case IPD is available. IPD-Analyses are used to answer specific questions, e.g. on subgroups, to adjust for confounding factors and to increase the power of detecting treatment effects and sources of heterogeneity [9,13,14]. In the case of IPD in meta-analysis, data from individual studies is modeled simultaneously within an appropriate specification of the meta-analysis as for example a random effects model [13].

If there is no evidence available from direct studies, unadjusted indirect comparisons can be performed after applying propensity score matching at the patients in the treatment arms coming from different studies in order to overcome the problem of neglecting the randomization of the individual studies. As a general limitation of this approach the treatment arms of the matched population may be only comparable with respect to the known confounders used for matching. An additional limitation is that a suitable matching partner is not in general available for every patient– by exclusion of these patients a new thread of bias can arise.

#### Underlying assumptions of indirect comparisons

The three basic assumptions to use adjusted indirect and mixed treatment comparisons appropriately are [1,2,7,15]:

- Homogeneity of results (assumption of common meta analyses),
- Similarity (comparability of the studies with regard to effect modifiers) and
- Consistency (comparability of the estimated effect from direct and indirect evidence).

**Figure 3:**
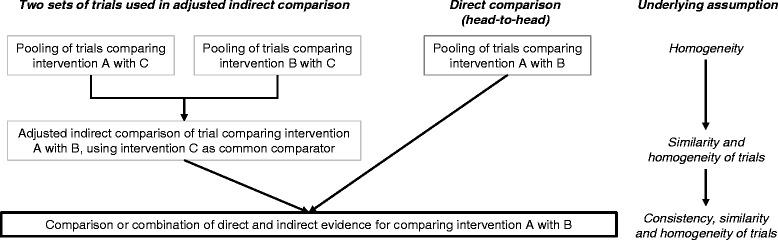
**Assumptions underlying adjusted indirect and mixed treatment comparison.** Assumptions of the figure are based on a single common comparator. Source: [16], adapted.

The analysis of the assumption on similarity can be carried out by subjective evaluation of the study characteristics and can be supplemented by sub-group analyses or meta-regression, if necessary. However, statistical methods to evaluate the assumption on consistency are currently being developed. There are still many open methodological questions in this area [16]. The underlying assumptions of indirect mixed treatment comparisons are shown in Figure 3.

### IQWiG and G-BA requirements in Germany [7]

In Germany, the IQWiG generally recommends adjusted indirect comparisons (particularly the approach by Bucher [4]) and usually rejects unadjusted indirect comparisons as an invalid analytical method. Due to current unsolved methodological problems IQWiG also recommends not using MTC within benefit assessments. The underlying methodology of an indirect comparison which is used in a benefit dossier has to be consistent (regarding comparability of effects) and must be described very accurately to allow a reproduction of the procedure by IQWiG and G-BA [7,15].

### Requirements for indirect comparisons in other European countries

#### France [17]

The Haute Autorité de Santé (HAS) accepts adjusted indirect comparisons as a valid method of indirect comparisons. As an inappropriate approach, the naïve comparison of active arms and the naïve comparison of point estimates are named. If a direct comparison is possible, HAS states that even then an indirect comparison could be useful. A mixed approach (MTC) of direct and indirect comparisons can be used to assess the validity of the results of an adjusted indirect comparison. HAS suggests the use of the following estimates for networks: Bayesian network meta-analysis (model of Lu/Ades and Caldwell) and Lumley’s network meta-analysis (mixed linear model) [17].

#### *England* [18]

National Institute for Health and Care Excellence (NICE) recommends to present data from a MTC (includes adjusted indirect comparisons) if no direct evidence (head-to-head trials) is available. The MTC should incorporate RCTs comparing at least two of the relevant (intervention or comparator) treatments. It is required to present a detailed and complete description of the MTC. In adjusted indirect comparisons and MTC the principals of good practice for standard pairwise meta-analyses have to be followed [18].

#### Scotland

It is to assume that Scottish Medicines Consortium (SMC) leans the acceptance of indirect comparisons within health technology assessments on the requirements of NICE. A checklist for manufacturers is provided by SMC to assess the indirect comparison which was made in the HTA.

A comparison of the acceptance of the different methods of indirect comparisons of IQWiG, HAS, NICE and SMC is shown in Table 1.

**Table 1:** **Acceptance of methodology of indirect comparisons by IQWiG, HAS, NICE and SMC within benefit assessments**

**Methodology of indirect comparison**	**Explanation**	**Acceptance by**			
		**IQWiG**	**HAS**	**NICE**	**SMC**
Unadjusted	•Naïve use of single study arms of different trials (violation of randomization)	−	−	−	/
	•Application of matching procedures and multivariate analysis				
Adjusted	•Meta-analytical summary of RCT outcomes	+	+	+	/
	•Subtraction of realized therapeutic effects (control group vs. verum group(s))				
	•Comparison of adjusted verum groups				
MTC	•Combination of evidence from direct and indirect comparisons	−	+	+	/
	•Basic assumptions of adjusted indirect comparison are applied				
	•Efficacy ranking of examined therapies				
	•Bayesian and frequentist approach				

+: yes, −: no, /: unknown.

Sources: [7,17,18].

In addition to the HTA-agencies within Europe, a new collaboration was created by them to provide guidance within HTA in Europe: the European Network for Health Technology Assessment (EUNETHTA).

#### *EUNETHTA Guidance* [6]

According to the EUNETHTA Guidance, adjusted indirect comparisons preserve randomization within trials and should always be used in preference to unadjusted methods. Accepted methods are Bucher’s method of adjusted indirect comparisons, Bayesian and Lumley’s method of MTC. Recently Bayesian MTC replaced more and more adjusted indirect comparisons according to Bucher. Compared to Bucher’s method, Bayesian MTC can analyze more complex evidence networks and include more study-level covariates than Bucher [6].

## Methods

The aim of this study was to evaluate the relevance of indirect comparisons in the German early benefit assessment of new drugs and other HTA processes in Europe. Therefore the submissions of indirect comparisons assessed by IQWiG within the time frame of January 2011 until February 2014 were comprehensively analyzed.

First, all submissions in Germany were analyzed, to identify the new drugs within the defined time frame where submissions contained indirect comparisons. Second, for each indirect comparison it was determined which method was applied. Third, the decisions of IQWiG were analyzed whether the indirect comparison was accepted or rejected and reasons for rejection were identified and classified in formal or methodological deficiencies.

Additionally, a systematic comparison of all German indirect comparisons with submitted and assessed comparisons in different European countries was performed. For this comparison, three European countries with a comparable and established HTA-process to Germany and a minimal level of transparency that allows a comparison were chosen: France (HAS), England (NICE) and Scotland (SMC).

## Results

### Indirect comparisons submitted to IQWiG

Of all submitted dossiers from manufacturers to IQWiG until February 2014, 23 indirect comparisons could be identified. Of these 23 indirect comparisons submitted by manufacturers the comparison of choice is the adjusted indirect comparison (n = 14) as required by IQWiG methods. MTC were submitted in four cases, mostly with a Bayesian approach. Only five out of 23 were unadjusted indirect comparisons as shown in Table 2. Reasons for rejection of indirect comparisons by IQWiG can be classified in either formal or methodological deficiencies.

**Table 2:** **Evaluation of method used within indirect comparisons submitted to IQWiG and reasons for rejection**

**Drug substance**	**Trade name**	**Method used for indirect comparison**		**Reasons for rejection by IQWiG**	
Abirateron acetat	Zytiga	Adjusted indirect comparison	Bucher’s method, pair wise	•Comparator	•Incompleteness of the used study pool
					•Inclusion criteria of the bibliographic search and data used for each population remained unclear
				•Formal	•Different appropriate comparator as determined by G-BA
Aclidinium bromide	Eklira	MTC	Bayesian approach	•Formal	•Data was not presented adequately (outcomes differ from original source, lacking traceability of data, inadequate inclusion of studies etc.)
Aflibercept	Eylea	Unadjusted indirect comparison	Descriptive	•Methodology	•Comparison does not fulfill the requirements of an adjusted indirect comparison
Axitinib	Inlyta	Unadjusted indirect comparison	simulated treatment comparison	•Methodology	•Comparison does not fulfill the requirements of an adjusted indirect comparison
				•Formal	•Description of the simulated comparison (documented in a
					•Program code) was missing
Collagenase clostridium histolyticum	Xiapex	Unadjusted indirect comparison	-	•Formal	•Comparison does not fulfill the requirements of an adjusted indirect comparison
					•Missing common comparator
				•Methodology	•Patient population was not in line with the application population
Dapagliflozin^a^	Forxiga	Adjusted indirect comparison	Bucher’s method, frequentist approach	•Comparator	•Methodological mismatch: study population within the studies used for comparison was not the same as the indication population
				•Methodology	•Different appropriate comparator as determined by G-BA
Fingolimod	Gilenya	MTC	Bayesian approach	•Methodology	•Inconsistencies in the study search and an inadequate population
				•Formal	
Ingenolmebutat	Picato	Unadjusted indirect comparison	-	•Methodology	•Comparison does not fulfill the requirements of an adjusted indirect comparison
					•Common comparator was missing
Linagliptin	Trajenta	Adjusted indirect comparison	Bucher’s method	•Comparator	•Different appropriate comparator as determined by G-BA
Perampanel	Fycompa	Adjusted indirect comparison	Bucher’s method	•Comparator •Methodology	•Different appropriate comparator as determined by G-BA •Examination of a part of the population was criticized
Retigabine	Trobalt	MTC	Frequentist approach	•Comparator	•Different appropriate comparator as determined by G-BA
Ticagrelor^a^	Brilique	Adjusted indirect comparison	Bucher’s method, frequentist approach	-	•Missing validity of endpoints, quality of trials and evidence
					•Methodological restrictions for simple adjusted indirect comparison
Telaprevir	Incivo	MTC	Bayesian approach	•Comparator	•Different appropriate comparator as determined by G-BA
				•Methodology	•Adequate data for handling subgroups was missing (interaction test)
Dabrafenib	Tafinlar	Adjusted indirect comparison	Bucher’s method	•Comparator	•Different appropriate comparator as determined by G-BA
Elvitegravir, Cobicistat, Emtricitabin, Tenofovirdisoproxil^a^	Stribild	Adjusted indirect comparison	Frequentist approach	•Methodology	•Patient population and applied transferability of patient data cannot be followed
Fampridin	Fampyra	Unadjusted indirect comparison	-	•Comparator	•Comparison does not fulfill the requirements of an adjusted indirect comparison
					•Patients, who did not receive best supportive care (physiotherapy) are not similar to patients with placebo treatment (Placebo as common comparator)
				•Methodology	•No data considered with appropriate comparator
Lixisenatid	Lyxumia	Adjusted indirect comparison	Bucher’s method, pair wise	•Comparator	•Different appropriate comparator as determined by G-BA in one indication
				•Methodology	•Differences in patient population, common comparator and application of comparators is not as authorized within included trials
Saxagliptin	Onglyza	Adjusted indirect comparison	Frequentist approach	•Methodology	•Use of inadequate patient population and study period
					•Differences in used common comparators
Saxagliptin (new indication)	Onglyza	Adjusted indirect comparison	Frequentist approach	•Comparator	•Different appropriate comparator as determined by G-BA
Saxagliptin/Metformin (new indication)	Komboglyze	Adjusted indirect comparison	Frequentist approach	•Methodology	•Use of inadequate patient population and study period
					•Application of comparators is not as authorized within included trials
Sitagliptin	Januvia, Xelevia	Adjusted indirect comparison	Bucher’s method, pair wise	•Methodology	•Use of inadequate patient population and study period
					•Application of comparators is not as authorized within included trials
				•Formal	•Missing sensitivity analyses
Teriflunomid	Aubagio	Adjusted indirect comparison	Bucher’s method, MTC	•Methodology	•Incomplete study pool
				•Formal	•Heterogeneity of included studies and non consideration of heterogeneity within indirect comparison
Vildagliptin^a^	Galvus, Jalra, Xiliarx	Adjusted indirect comparison	Bucher’s method	•Methodology	•Use of inadequate patient population and study period
					•No statement towards authorization conform patient population
					•Differences in used common comparators

^a^Indirect comparison not in all submitted indications.

Sources: [19-78].

#### Formal deficiencies

Formal deficiencies are defined as a mismatch of the applied method and the method required by IQWiG and G-BA. Within formal deficiencies, different reasons for the rejection of an indirect comparison by IQWiG could be identified: choice of an appropriate comparator different to the one determined by the G-BA, formal deficiencies related to the bibliographic search, formal deficiencies related to the indirect comparison and incomplete documentation.

One main reason for the rejection of indirect comparisons by IQWiG is the choice of an appropriate comparator which differs from the one determined by G-BA. The choice of an appropriate comparator by the manufacturer different to the one determined by G-BA is legitimate if justified adequately. In all ten cases the justification of a different choice has been declined and the indirect comparisons (adjusted indirect comparisons or MTC) were rejected due to an inappropriate standard therapeutic approach (Table 2). This was the case for Abiraterone acetate, Dapagliflozin, Linagliptin, Perampanel, Retigabine, Telaprevier, Dabrafenib, Fampridin, Lixisenatid and Saxagliptin (label extension).

As formal deficiencies related to the bibliographic search, the incompleteness of the used study pool and an unclear definition of inclusion criteria for bibliographic search and data used for each population (Abirateron acetat) as well as inconsistencies in the search within study registries (Fingolimod) were criticized.

As formal deficiencies related to the indirect comparison, missing sensitivity analysis and a non authorized application of the comparator within included trials were criticized in the indirect comparison of Sitagliptin.

As incomplete documentation, the required program code of the indirect comparison (simulated treatment comparison) was missing in the case of Axitinib (When an indirect comparison is submitted within the German benefit assessment, it is required for the manufacturer to provide the program code in an attachment (Module 5)).

#### Methodological deficiencies

Methodological deficiencies are defined as an inappropriate methodology of the indirect comparison, as for example heterogeneity of study results within MTC, non-similarity of studies, inconsistencies of outcomes of comparisons. Five indirect comparisons of Aflibercept, Axitinib, Collagenase clostridium histolyticum, Ingenolmebutat and Fampridin were rejected by IQWiG due to the use of an unadjusted indirect comparison (Table 2), which did not fulfill the requirements of an adjusted indirect comparison.

In addition, the common comparator was missing or differing in the indirect comparisons of Ingenolmebutat, Fampridin, Lixisenatid, Saxagliptin and Vildagliptin. An inadequate or heterogenic patient population and/or differing study periods which were included in the comparison were criticized for Elvitegravier, Cobicistat, Emtricitabin, Tenofovirdisoproxil, Fampridin, Lixisenatid, Saxagliptin, Saxagliptin/Metformin, Sitagliptin and Vildagliptin. A mismatch in the included indications was another critique from IQWiG for Dapagliflozin. The heterogeneity of included studies and the missing consideration of heterogeneity within the indirect comparison were also criticized by IQWiG (Teriflunomid).

In summary the most common rejection reason by IQWiG of an indirect comparison is a differing appropriate comparator as determined by G-BA as well as a patient population (pivotal trial-population) which differs from population defined in the indication according to the summary of product characteristics. Rejections due to true formal deficiencies are rare, as none adequately presented data or description of the comparison as shown in Table 2.

### Comparison to HTA processes in England, France and Scotland

For the same new drugs, manufacturers submitted six indirect comparisons to HAS, five to NICE and 12 to SMC. As shown in Tables 1 and 3, there are differences in the submitted comparisons as well as HTA-dossiers and available transparency (submitted dossiers, assessment by HTA-agency etc.) between each country. Additionally, a dossier including an indirect comparison was not always submitted to the respective HTA-agency. A comparison between France and Germany is difficult, due to discrepancies between submitted dossiers regarding drug substance and applied indirect comparisons, which mostly differ in each country. In England additional assessments are performed for new products, besides HTA. Therefore a comparison of dossiers with indirect comparisons is challenging as with block scoping reports, policy statements and guidelines the submission of a dossier is not mandatory for manufacturers. For Scotland, most decisions by SMC are not transparent regarding the acceptance of the indirect comparison submitted by the manufacturer.

**Table 3:** **Evaluation of indirect comparisons submitted to IQWiG, HAS, NICE and SMC**

**Drug substance**	**Trade name**	**Acceptance of indirect comparison**			
		**Germany**	**France**	**England**	**Scotland**
Abirateron acetat	Zytiga	No	**Yes**	-	-
Aclidinium bromide	Eklira		No		
Aflibercept	Eylea		-	No^e^	**Yes**
Axitinib	Inlyta		No	Ongoing	Unclear
Collagenase clostridium histolyticum	Xiapex		-	-	
Dapagliflozin^a^	Forxiga			No	
Fingolimod	Gilenya				
Ingenolmebutat	Picato			-	-
Linagliptin	Trajenta				Unclear
Perampanel	Fycompa				
Retigabine	Trobalt			**Yes**	**Yes**
Ticagrelor^b^	Brilique	Yes	**No**	-	-
Telaprevir	Incivo	No	-		
Dabrafenib	Tafinlar			Ongoing	
Elvitegravir, Cobicistat, Emtricitabin, Tenofovirdisoproxil^a^	Stribild			-^c^	
Fampridin	Fampyra				
Lixisenatid	Lyxumia				Unclear
Saxagliptin	Onglyza				**Yes**
Saxagliptin (new indication)	Onglyza			-	-
Saxagliptin/Metformin (new indication)	Komboglyze				
Sitagliptin	Januvia, Xelevia		Ongoing	-^c^	Unclear
Teriflunomid	Aubagio		-	No	-
Vildagliptin^a^	Galvus, Jalra, Xiliarx			-^c^	Unclear^d^

^a^Indirect comparison not in all submitted indications.

^b^Indirect comparison only for STEMI PIC population.

^c^No HTA report available due to existence of block scoping report, policy statement, guideline, recommendation and evidence summary.

^d^Indirect comparison was used as sensitivity analysis to support the primary analysis.

^e^Indirect comparison of included trials should be taken with caution, due to a high heterogeneity of these trials.

The detailed reasons of rejection of the comparisons in Germany are presented in Table 2.

Sources: [17,19-121].

When comparing the submitted dossiers that involved an indirect comparison in Germany (IQWiG) with those submitted in France (HAS), England (NICE) and Scotland (SMC), differences in the assessments of Abirateron acetat (Zytiga®), Aflibercept (Eylea®), Retigabine (Trobalt®), Ticagrelor (Brilique®) and Saxagliptin (Onglyza®) were revealed:

#### Abirateron acetat (Zytiga®) [55,115]

IQWiG decided opposite of HAS against the submitted indirect comparison of Abirateron acetat. In Germany, the manufacturer submitted an indirect comparison with a paired comparison according to Bucher’s method and Prednison as common comparator. In France, there are no information or specifications available regarding methodology or common comparator used. In both dossiers Abirateron acetat was compared with Cabazitaxel. However, IQWiG did not accept this comparison due to the fact that Cabazitaxel was not the appropriate comparator which had been chosen by the G-BA.

#### Aflibercept (Eylea®) [66,98]

In Scotland the indirect comparison of Aflibercept was accepted by SMC with limitations. The limitations were applied due to a small number of included trials, heterogeneity between trials and patient baseline characteristics. Three types of indirect analyses were submitted by manufacturer in Scotland: a Bucher analysis, a Bayesian network analysis (both random effects) and a frequentist network analysis (fixed effects). In change, an unadjusted indirect comparison (which was described as a descriptive indirect comparison by the pharmaceutical company) has been submitted in Germany. Also a common comparator was missing in the German submission as well as an appropriate comparator which was not used as licensed (within trials). Due to this, the submitted German indirect comparison was not accepted by IQWiG.

#### Retigabine (Trobalt®) [59,79,88]

In the dossier submitted for NICE, a MTC with a Bayesian approach was conducted and Placebo was used as common comparator. The comparison was made between Retigabine and different anticonvulsants, e.g. Lacosamide, Pregabalin, Zonisamide. The manufacturer submitted an indirect comparison with Placebo as common comparator and a comparison with Eslicarbazepin and Lacosamid to the SMC. In the contrary, an indirect comparison with dose specific (pair wise comparison with fixed and random effects) and dose unspecific (pooled estimates) meta-analyses was submitted in Germany and failed the IQWiG assessment. As well as in England and Scotland, Placebo was the common comparator. The comparison was made between Retigabine and Lacosamid, which did not correspond to the appropriate comparator (Lamotrigine and Topiramat) chosen by G-BA.

#### Saxagliptin (Onglyza®) [72,103]

In the SMC-submission, the manufacturer presented two adjusted indirect comparisons, using the simple Bucher method and included three studies with Placebo as common comparator. In comparison the German submission included four indirect comparisons for four indications with different common comparators. An adjusted indirect comparison (frequentist approach) was conducted in all four indications and declined by IQWiG as the chosen comparators did not correspond to the appropriate comparators chosen by the G-BA.

#### Ticagrelor (Brilique®) [61,110]

In contrast to the positive assessment of the indirect comparison for the STEMI-population of Ticagrelor by IQWiG, HAS did not accept the indirect comparison which was submitted within the dossier. As far as reproducible both dossiers (German and French) included the same trials (PLATO and TRITON) as well as the same common comparator (Clopidogrel). In Germany a simple adjusted indirect comparison with a frequentist approach was conducted. In France the specific method was not described.

In summary, all five indirect comparisons are not comparable between Germany and France due to different submitted comparisons and a diverging level of public transparency.

## Discussion

### Rejections by IQWiG

In the first year of the benefit assessments there were more formal deficiencies compared with the last 12 months of this analysis. As an improvement could be noted that data is usually presented adequately (e.g. Aclidinium bromide) and program codes normally are presented (e.g. Axitinib) by manufacturers. However, the most frequent methodological deficiency is still the application of a comparator differing from the appropriate comparator defined by G-BA.

In this case benefit assessments were fully declined and not further evaluated even if the indirect comparison was adequate and methodologically correct according to IQWiG (e.g. Linagliptin, Retigabin).

One of the biggest challenges for manufacturers within the German AMNOG assessment is the MTC – as validity is depending on the method used. This means the assumptions on similarity (comparability of the studies with regard to effect modifiers) and, in case of a combination of direct and indirect evidence, the assumptions on consistency (comparability of the estimated effect from direct and indirect evidence) have to be fulfilled. The use of the relevant population, interventions and inclusion of relevant outcomes as well as a high level of evidence (RCT) are some of the requirements for a successfully accepted MTC. But conflicts occur within patient populations (trial population vs. indication and authorized subpopulation; e.g. Perampanel), duration of trials (long vs. short; e.g. Teriflunomid) and treatment regimens (authorization vs. trial usage; e.g. Saxagliptin/Metformin). Also, even with the use of only randomized trials within MTC validity is at risk due to biases.

As the analysis shows, the rejection of indirect comparisons within HTA assessments is very frequent in Germany. However, a comparison with other European HTA-agencies does not allow a comparative analysis, and only reflects a discrepancy within transparency of data and availability of reports.

### Transparency within Europe

To allow a comparison of the different requirements and acceptance of indirect comparisons across Europe, more transparency is necessary. It is difficult to perform an objective comparison due to the discrepancy of available and feasible public data. Only in Germany, the manufacturer’s dossier, HTA assessment, resolution and justifying reasons are fully available for the public. In addition the HTA assessment is very detailed and allows a recapitulation by third parties, which is not always possible in other countries. Due to that a comparison of HTA-decisions across Europe for new pharmaceutical products is not feasible.

### Methodology within Europe

Within Europe the methodological requirements for an indirect comparison are similar, as the gold standard is an adjusted indirect comparison in Germany, France, England and Scotland (Table 3). However, IQWiG is more hesitant in accepting new methods as MTC compared with HAS, NICE and SMC due to open methodological questions.

One of the major differences within European HTA agencies is the rejection of indirect comparisons due to a differing appropriate comparator as determined by G-BA in Germany, which is not known as current practice in England and Scotland. This is the main reason why an evaluation of indirect comparisons across Europe is not feasible. Also, no coherent policy for the acceptance of indirect comparisons (i.e. none acceptance of unadjusted indirect comparisons) within HTA-agencies across Europe could be identified. Rather, different pre-requisites and expectations have to be met across Europe when presenting evidence based on indirect comparisons.

### Limitations of indirect comparisons

In case of limited availability of studies or high heterogeneity between the available studies, no MTC or indirect comparisons should be performed. In case no common comparator is available an unadjusted indirect comparison remains as an option. Unadjusted indirect comparisons however lead to a higher variety of outcomes and uncertainties which limit the interpretation of the results [5].

The validity of a MTC depends on the method: were unadjusted or adjusted indirect comparisons conducted? MTC performed with an unadjusted indirect comparison lead to a higher bias potential and an over- or underestimation of therapeutic outcomes due to loss of randomization and structural homogeneity [5].

Within indirect comparisons methodological problems often occur due to the understanding of underlying assumption, in used methods as well as fixed and random effects, in the assessment of trial similarity (heterogeneity), in the comparison and combination of evidence as well as within publication biases [6,16].

### Political implications

The presented analysis of the status quo of the acceptance of indirect comparisons in Germany suggests a high probability of political implication at the choice of the appropriate comparator. As the appropriate comparator is most often the cheapest available product (i.e. generic or reference price) it has to be assumed that cost containment is one of the main drivers for the decision. The same conclusion was drawn by another publication of indirect comparisons which confirms that the German benefit assessment is depending on the additional benefit of a new product as well as on the decision of the G-BA for an appropriate comparator [122].

## Conclusion

The number of 23 indirect comparisons submitted between January 2011 and February 2014 shows that evidence for direct comparisons is often not available, so manufacturers have to perform indirect comparisons to compare their drugs with the appropriate comparator defined by G-BA. This shows the high importance of indirect comparisons within the German early benefit assessment. However, Ticagrelor is the only accepted indirect comparison in one subpopulation until now. It was also the first dossier which was assessed by IQWiG and received no additional benefit in this subpopulation. All other indirect comparisons were rejected by IQWiG due to methodological and formal deficiencies. So, the relevance of indirect comparison in the German benefit assessment is very limited as almost all indirect comparisons have been rejected so far.

At this point, it also has to be stated that the IQWiG approach is methodologically accurate and in accordance with the legal framework. However, a different appropriate comparator than chosen by the G-BA is mostly declined in the assessment with the consequence of rejection of the indirect comparison and no additional benefit. This could lead to the question, if the currently applied methodology to determine the appropriate comparators is useful and feasible. Based on that indirect comparisons are generally accepted within the German early benefit assessment, the challenge for the future will be to bring together: the determination of the appropriate comparator by G-BA and the selection of the appropriate comparator, study choice and selection of patient subgroups by the manufacturer to give indirect comparisons a higher relevance in Germany.

Despite of the German-specific implications, a comparison of the relevance of indirect comparison in the German early benefit assessment with HTA processes in England, France and Scotland is not feasible due to differential submitted comparisons and a diverging level of public transparency. To compare and assess submitted indirect comparisons it would be desirable to establish a transparent process and a mandatory publication of HTA-reports within Europe. This is necessary to be able to learn of methodological deficiencies and acquire new methods to address heterogeneity and biases within indirect comparisons.

First approaches to assimilate methodological requirements of indirect comparisons within Europe are in progress as the European guideline from EUNETHTA and the recently published report of the ISPOR-AMCP-NPC Good Practice Task Force show [6,123]. But it is not enough to develop valid methodological approaches to solve heterogeneity issues and reduce biases only. International guidelines for Europe accepted by national HTA-agencies would be desirable.

To avoid the need of indirect comparison, manufacturers could encourage with the HTA agencies to set up study designs for Phase III trials that allow direct comparisons. But this would also imply that the different HTA agencies demand the same methodology and agree on the use of the same appropriate comparator. As already mentioned, in Europe first steps are taken but a common methodology is not yet established.

Overall, external preconditions and methodological requirements are demanding and hardly to fulfill. At present, manufacturers rely on the use of adequate methods (status quo), the right appropriate comparator and missing formal deficiencies to submit an indirect comparison which may be accepted by IQWiG – a methodological discussion with the current players in the system (manufacturer, IQWiG, G-BA and scientific leaders) is pending.
